# Nomogram for prediction of peritoneal metastasis risk in colorectal cancer

**DOI:** 10.3389/fonc.2022.928894

**Published:** 2022-11-07

**Authors:** Xian-qing Song, Zhi-xian Liu, Qing-yuan Kong, Zhen-hua He, Sen Zhang

**Affiliations:** ^1^ General Surgery Department, Ningbo Fourth Hospital, Ningbo, Zhejiang, China; ^2^ Proctology Department, Beilun People’s Hospital of Ningbo, Ningbo, Zhejiang, China; ^3^ General Surgery Department, Baoan People’s Hospital of Shenzhen, Shenzhen, Guangdong, China; ^4^ General Surgery Department, Hezhou People’s Hospital, Hezhou, Guangxi, China; ^5^ Department of Colorectal Surgery, First Affiliated Hospital of Guangxi Medical University, Nanning, Guangxi, China

**Keywords:** colorectal cancer, peritoneal metastasis, nomogram, LASSO, logistic regression analysis

## Abstract

**Objective:**

Peritoneal metastasis is difficult to diagnose using traditional imaging techniques. The main aim of the current study was to develop and validate a nomogram for effectively predicting the risk of peritoneal metastasis in colorectal cancer (PMCC).

**Methods:**

A retrospective case-control study was conducted using clinical data from 1284 patients with colorectal cancer who underwent surgery at the First Affiliated Hospital of Guangxi Medical University from January 2010 to December 2015. Least absolute shrinkage and selection operator (LASSO) regression was applied to optimize feature selection of the PMCC risk prediction model and multivariate logistic regression analysis conducted to determine independent risk factors. Using the combined features selected in the LASSO regression model, we constructed a nomogram model and evaluated its predictive value *via* receiver operating characteristic (ROC) curve analysis. The bootstrap method was employed for repeated sampling for internal verification and the discrimination ability of the prediction models evaluated based on the C-index. The consistency between the predicted and actual results was assessed with the aid of calibration curves.

**Results:**

Overall, 96 cases of PMCC were confirmed *via* postoperative pathological diagnosis. Logistic regression analysis showed that age, tumor location, perimeter ratio, tumor size, pathological type, tumor invasion depth, CEA level, and gross tumor type were independent risk factors for PMCC. A nomogram composed of these eight factors was subsequently constructed. The calibration curve revealed good consistency between the predicted and actual probability, with a C-index of 0.882. The area under the curve (AUC) of the nomogram prediction model was 0.882 and its 95% confidence interval (CI) was 0.845–0.919. Internal validation yielded a C-index of 0.868.

**Conclusion:**

We have successfully constructed a highly sensitive nomogram that should facilitate early diagnosis of PMCC, providing a robust platform for further optimization of clinical management strategies.

## 1 Introduction

According to the latest global cancer statistics in 2021, more than 1.9 million new colorectal cancer (CRC) (including anal) cases and 935,000 related deaths were recorded in 2020 from 185 countries, accounting for 10% of all 36 cancer types. Overall, CRC ranks third in terms of incidence and second in terms of mortality worldwide (1). In recent decades, the survival rate of metastatic CRC (mCRC) has improved owing to multidisciplinary discussions and provision of individualized comprehensive treatment regimens, in particular, molecular targeted therapy and biological immunotherapy. Molecular biomarkers such as RAS, BRAF and PIK3CA (key driver genes mutated in CRC) (2) and microsatellite instability (MSI) state have been successfully applied to guide targeted and immunotherapy decisions in clinical practice (3). However, treatment of mCRC remains a significant challenge, with local or distant recurrence commonly reported in numerous patients with stage II or III disease (4). Documented studies so far have reported that ~21% patients are diagnosed with distant dissemination (5) and >85% mCRCs do not have specific driver genes (6), especially colorectal cancer patients with peritoneal metastasis (PMCC), 4% of which are characterized by solitary peritoneal dissemination (7). Effective treatment options for PMCC in the clinic are limited at present. Peritoneum is the third common metastasis site after liver and lung (8) and PMCC is associated with poor survival rates and prognosis. In the past two decades, PMCC has been considered a local progressive disease and try to establish and explore the palliative treatment based on this concept. However, even after active medical intervention, median survival rate remains between 10 and 18 months (9). Moreover, since conventional imaging modalities such as computed tomography (CT) lack spatial resolution to effectively detect early peritoneal diseases and tumor markers are usually the only available tool for diagnosis and evaluation of therapeutic effects, rapid and early identification these patients remain a major challenge. The overall sensitivity of CT scanning in PM is 43%, sensitivity to lesions >5 mm is 94% and that to lesions <5 mm is reduced to 11% (10). Existing studies showed that the sensitivity and specificity of PET-CT for PMCC were 85% and 88%, respectively (11). However, PET-CT is also constrained by lesions with a diameter less than 1 cm. The uptake rate of 18F-fluorodeoxyglucose (18F-FDG) by certain pathological subtypes, particularly those prone to peritoneal metastasis formation (i.e., poorly differentiated adenocarcinomas and mucinous adenocarcinomas) is not high, affecting the diagnostic value of PET-CT scanning in the detection of these lesions (12).Therefore, modern imaging techniques are unable to effectively detect peritoneal metastasis at the early stages. Additionally, current non-invasive assessments (such as clinical examination, imaging or biology) are ineffective. At present, the gold standard for PM assessment is early detection through systematic surgical exploration (diagnostic laparoscopy or laparotomy). While surgical exploration displays greater sensitivity in diagnosis of PMCC, the procedure is invasive and expensive, along with significant risk of surgical complications (13). Therefore, this method cannot be recommended for all relevant patients and is only employed for high-risk peritoneal metastasis cases. Identification of more reliable tools for early prediction of risk of PMCC is essential for early intervention and improvement of outcomes.

The nomogram is an effective prediction tool that can quantify risk using statistical software combined with all known risk factors and has been practically applied for diagnosis of several diseases. To date, relatively few nomograms have been developed to predict peritoneal metastasis risk in colorectal cancer patients. For many cancer types (14–19), nomograms show better performance than the traditional TNM staging system and are therefore recommended as an alternative method or even a new prediction standard for diagnosis of recurrence and metastasis for various tumor types. In view of the unreliability of imaging, clinical and biological tests, we constructed a nomogram for prediction of PMCC as a guide for patient management in this study. Our newly developed nomogram provides more personalized prediction criteria that should aid in optimization of management decisions for PMCC.

## 2 Materials and methods

### 2.1 Ethics statement

All patients provided written informed consent for information storage in the hospital database of the First Affiliated Hospital of Guangxi Medical University. We obtained separate consent for the use of this information for our research. Study approval was obtained from the independent Ethics Committee of the First Affiliated Hospital of Guangxi Medical University. Our research was performed in accordance with the ethical standards of the World Medical Association Declaration of Helsinki. Patients did not receive economic compensation.

### 2.2 Inclusion and exclusion criteria

Inclusion criteria were as follows: (a) patients were over 18 years of age, (b) tumor resection was performed, (c) no peritoneal metastasis was detected with preoperative CT or other imaging examinations and postoperative histological examination confirmed colorectal cancer metastasis, (d) primary colorectal cancer was confirmed with histopathology and peritoneal dissemination was synchronous, and (e) complete preoperative imaging and serological data were available. Exclusion criteria were as follows: (a) patients were younger than 18 years of age, (b) imaging and serological data were incomplete or unavailable, (c) patients were diagnosed with mental disorders or severe liver and kidney dysfunction, (d) history of neoadjuvant chemotherapy, and (e) detection of other tumor types at the time of diagnosis or history of cancer. Based on the above criteria, we included 1284 consecutive patients with colorectal cancer who underwent surgery in the First Affiliated Hospital of Guangxi Medical University from January 2010 to December 2015 for case analysis, model construction and internal validation.

### 2.3 Clinicopathologic variables

In this retrospective case-control study, the clinical data collected included sex, age, blood group, course, race, initial symptoms, tumor location, perimeter ratio, tumor size, liver metastasis, lung metastasis, gross type, pathological type, pathological grade, tumor invasion depth, Dukes stage, T-stage, N-stage, M-stage, total protein level, albumin, and CEA level. According to the 8^th^ edition of Tumor-Node-Metastasis (TNM) staging guidelines for colorectal cancer (20) issued jointly by the American Joint Committee on Cancer (AJCC) and the Union International Center of Cancer (UICC), ctTNM staging, tumor invasion depth and Dukes staging were defined in combination with colonoscopy/pathological diagnosis and preoperative enhanced CT scan data. Tumor size, perimeter, pathological type and grade were comprehensively assessed *via* preoperative imaging and electronic colonoscopy. According to primary tumor location in the left colon, right colon and rectum and the left and right colons are distinguished by the middle transverse colon. The cutoff values of age and tumor size were derived from the receiver operating characteristic (ROC) curve. The longest diameter of tumors was taken as tumor size.

### 2.4 Statistical analysis

All data were analyzed using IBM SPSS Statistics (Version 20.0, IBM corp., New York, USA) and R version 4.1.2 (The R Foundation for Statistical Computing, Vienna, Austria). Measurements for clinical indicators were transformed into classified variables according to median values of each group and SPSS software applied to analyze the statistical characteristics of all variables. The LASSO regression algorithm was used to select risk factors with optimal predictive value of colorectal cancer patients. Cross-validation was applied to confirm the appropriate tuning parameters (λ) for LASSO regression analysis. Finally, the most significant features were screened with the LASSO algorithm. After selecting the characteristics of non-zero coefficients in the LASSO regression model, independent risk factors of PMCC were determined *via* multivariate logistic regression and the nomogram prediction model established by combining the characteristics selected in the LASSO regression model.

Using the ‘ rms ‘ package of R software to build the PMCC nomogram prediction model, the score of all risk factors was added, whereby the probability of the total score corresponding to the model represented the probability of predicting PMCC before surgery. The nomogram presented risk factors in a graphical form and the risk of peritoneal dissemination in single patients could be calculated based on accumulating points related to each risk factor. Therefore, a higher score signified higher risk of PMCC.

The bootstrap method was used for repeated sampling 1000 times for internal verification of the nomogram model and the consistency index (C-index) calculated to determine its efficiency of discrimination. The area under curve (AUC) and calibration curve under receiver operating characteristics (ROC) (equivalent to C-index) were employed to evaluate the effectiveness and discrimination ability of the nomogram. ROC curve is a tool that can be used to graphically identify the cut-off value of any disease. AUC values ranged from 0 to 1, whereby 1 signified complete consistency. Values closer to 1 were indicative of stronger discrimination and prediction ability. In general, AUC values of 0.5–0.7 indicate low prediction ability, 0.7–0.9 medium prediction accuracy, and >0.9 high prediction accuracy. Differences were considered statistically significant at P < 0.05.

## 3 Results

### 3.1 Analysis of PMCC risk factors

#### 3.1.1 LASSO and logistic regression of colorectal cancer patients in the development set

**Figure 1:**
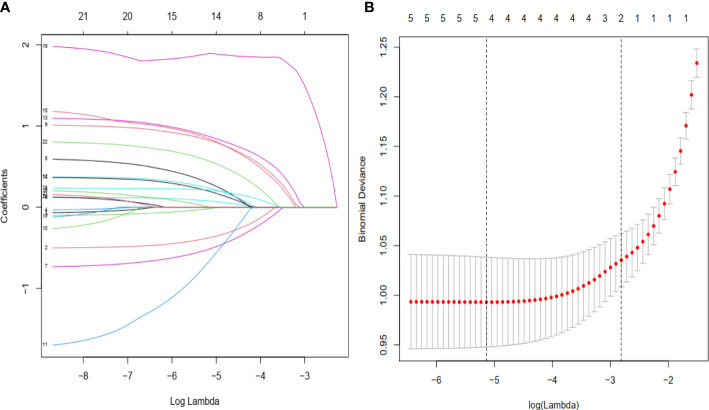
Demographic and clinical feature selection using the LASSO binary logistic regression model. **(A)** Twenty-two characteristic profiles of the LASSO coefficient. According to the logarithmic (lambda) sequence, a coefficient profile was generated. The optimal lambda produced 15 non-zero coefficients. **(B)** The optimal parameter (lambda) in the LASSO model was selected *via* 10-fold cross-validation using minimum criteria. The partial likelihood deviation (binomial deviation) curve relative to log (lambda) was plotted. A virtual vertical line at the optimal value was drawn using one SE of minimum criterion (the 1-SE criterion).

A total of 1284 patients with colorectal cancer were included, of whom 1188 (77.5%) showed no peritoneal metastasis in postoperative pathological examination and 96 (22.5%) had peritoneal metastasis. The demographic and clinical characteristics of patients in the study group are shown in the **Table 1**. In the LASSO regression model, 15 potential predictors with non-zero coefficients were selected from 22 features, including age, blood group, initial symptoms, tumor location, perimeter ratio, tumor size, lung metastasis, tumor gross type, pathological type, pathological grade, tumor invasion depth, Dukes’ stage, N-stage, M-stage, and CEA level, which could serve as risk factors for PMCC (**Figure 1**). We further used the ‘rms’ package in ‘R’ software to incorporate these clinicopathological factors into the logistic regression model for multivariate analysis. Ultimately, age (P = 0.024), tumor location (P = 0.002), perimeter ratio (P = 0.017), tumor size (P = 0.002), pathological type (P = 0.000), tumor invasion depth (P = 0.001), CEA level (P = 0.005) and gross type (P = 0.037) were identified as independent risk factors for PMCC (**Table 2**).

**Table 1:** Characteristics of patients included in this study.

		PMCC (n)		Incidence of PMCC (%)	X^2^/Z value	P value
		No	Yes			
Sex	Male	679	61	8.24	1.484	0.223
	Female	509	35	6.43		
Duration, month	<6	713	66	8.47	2.839	0.092
	≥6	475	30	5.94		
Tumor size(cm)	≤5cm	815	33	3.89	46.401	0.000
	>5cm	373	63	14.45		
Perimeter ratio	<1/2	496	24	4.62	10.343	0.001
	≥1/2	692	72	9.42		
Liver metastasis	No	1035	39	3.63	140.367	0.000
	Yes	153	57	27.14		
Lung metastasis	No	1171	95	7.50	0.097	0.755
	Yes	17	1	5.56		
Total protein level(g)	<60	293	24	7.57	0.005	0.941
	≥60	895	72	7.45		
Albumin(g)	<40	1007	85	7.78	0.997	0.318
	≥40	181	11	5.73		
CEA-level (ng/ml)	<10	883	40	4.33	46.879	0.000
	≥10	305	56	15.51		
Age (years)	≤40	177	27	13.24	21.207	0.000
	≥40 OR ≤60	495	48	8.84		
	>60	515	21	3.92		
Blood group	A	288	31	9.72	6.403	0.094
	B	337	25	6.91		
	AB	78	10	11.36		
	O	484	30	5.84		
Race	Ethnic Han	919	71	7.17	0.642	0.725
	Zhuang	216	20	8.47		
	others	51	5	8.93		
Initial symptom	Bowel	733	29	3.81	45.722	0.000
	Abdominal	316	56	15.05		
	Both	139	11	7.33		
Tumor location	Left colon	315	40	11.27	26.964	0.000
	Right colon	286	35	10.90		
	Rectum	586	21	3.46		
Gross type	Mass type	297	29	8.90	43.425	0.000
	Ulcer type	651	23	3.41		
	Infiltration type	240	44	15.49		
Histological type	Adenocarcinoma	1071	59	5.22	76.222	0.000
	Mucinous carcinoma	90	33	26.83		
	Others	27	4	12.90		
Grade	I	170	7	3.95	14.627	0.001
	II	873	65	6.93		
	III	145	24	14.20		
Ducks’ stage	A	171	3	1.72	214.129	0.000
	B	401	8	1.96		
	C	456	15	3.18		
	D	160	70	30.43		
T stage	T1	22	0	0.00	37.284	0.000
	T2	242	3	1.22		
	T3	773	63	7.54		
	T4	151	30	16.57		
N stage	N0	618	21	3.29	57.804	0.000
	N1	408	36	8.11		
	N2	162	39	19.40		
Tumor invasion depth	Mucosal	271	2	0.73	57.935	0.000
	Serosa	802	63	7.28		
	Outside the serous layer	115	31	21.23		
M stage	M0	1026	28	2.66	198.666	0.000
	M1	161	68	29.69		

**Table 2:** Predictors in the risk nomograms for PMCC using multivariate logistic regression.

Intercept and variable	Prediction model		
	β	Odds ratio (95% CI)	P-value
Intercept			
Age	-0.924	0.397 (0.177-0.890)	0.024
Tumor location	-1.486	0.226 (0.102- 0.487)	0.002
Perimeter ratio	0.783	2.187 (1.166- 4.256)	0.017
Tumor size	0.877	2.403 (1.374- 4.247)	0.002
Pathological type	1.427	4.167 (2.028- 8.539)	0.000
Tumor invasion depth	3.309	27.365 (5.083-237.594)	0.001
CEA level	0.814	2.256 (1.275-4.009)	0.005
Gross type	0.720	2.054 (1.049-4.087)	0.037

**β** is the regression coefficient.

CI, confidence interval; CEA, carcinoembryonic antigen.

### 3.2 Establishment, verification and evaluation of the nomogram

#### 3.2.1 Development and internal validation of our nomogram model in prognostic prediction of PMCC

**Figure 2:**
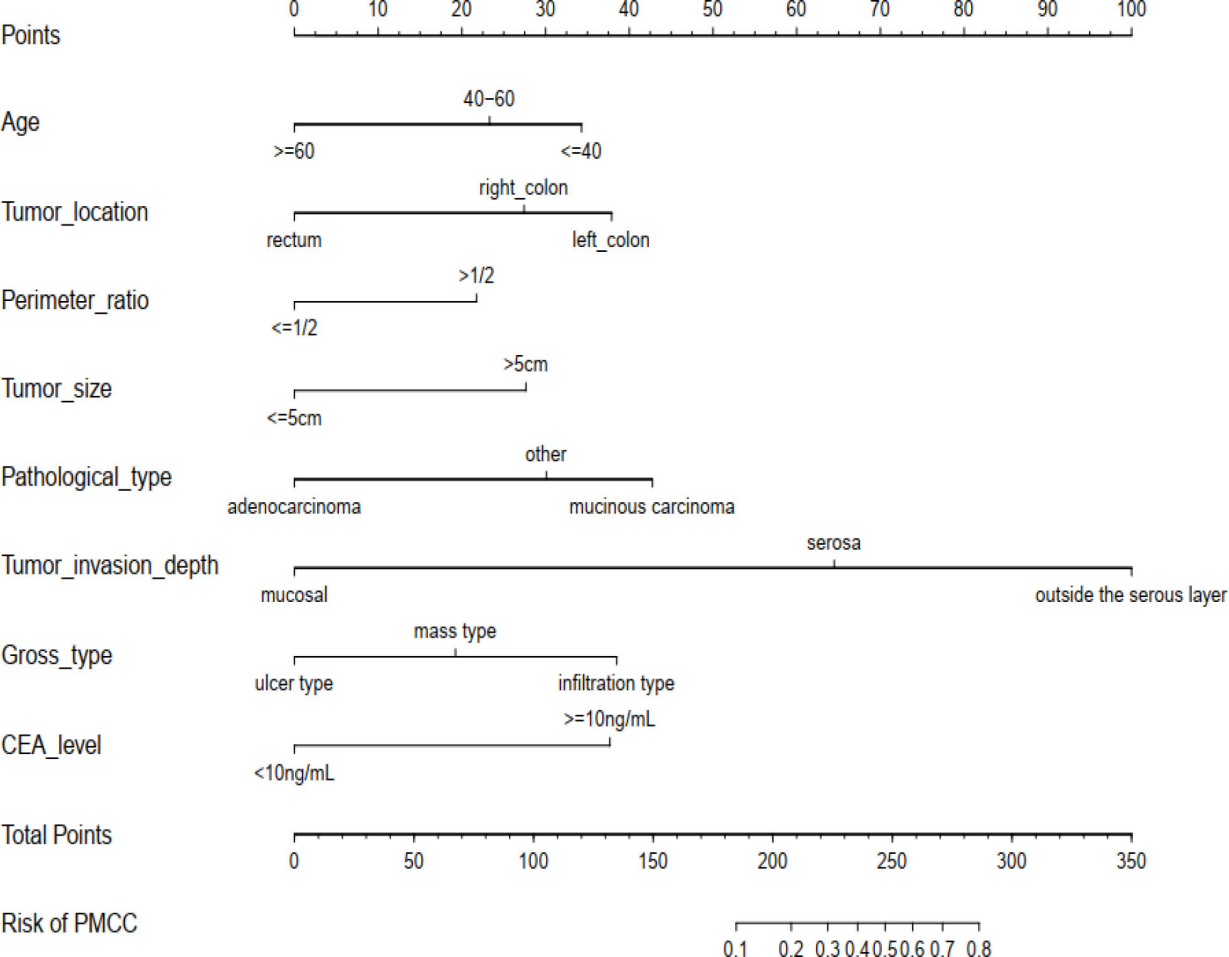
The newly developed PMCC prediction nomogram. PMCC, peritoneal metastasis of colorectal cancer; CEA, carcinoembryonic antigen.

**Figure 3:**
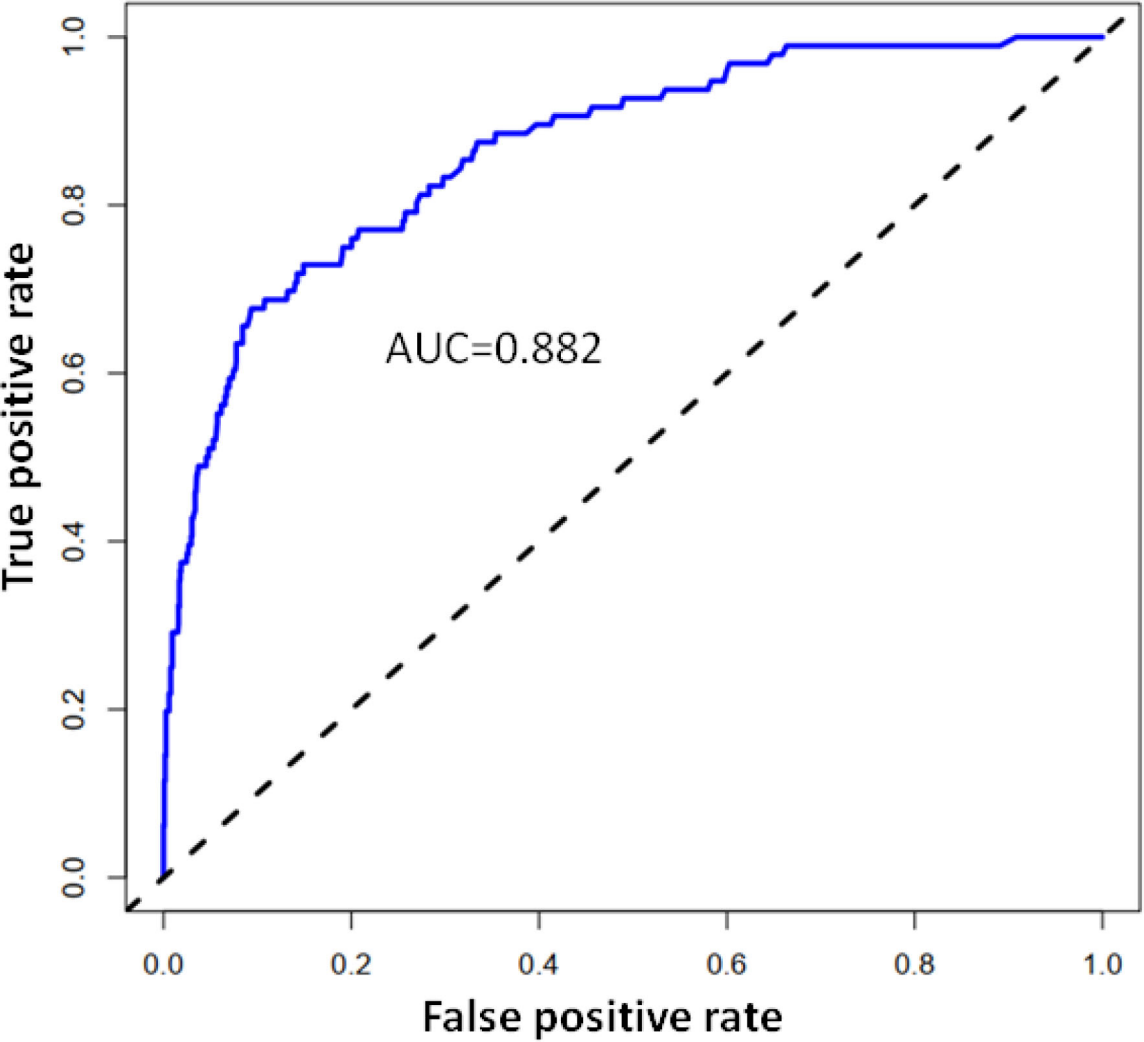
Calibration curve of the predictive value of the PMCC nomogram. The X-axis represents predicted PMCC risk and Y-axis represents actually diagnosed risk of PMCC. The diagonal dotted line represents the perfect prediction of the ideal model. The solid line represents the performance of nomogram (specifically, the closer to the imaginary diagonal line, the better the prediction effect).

**Figure 4:**
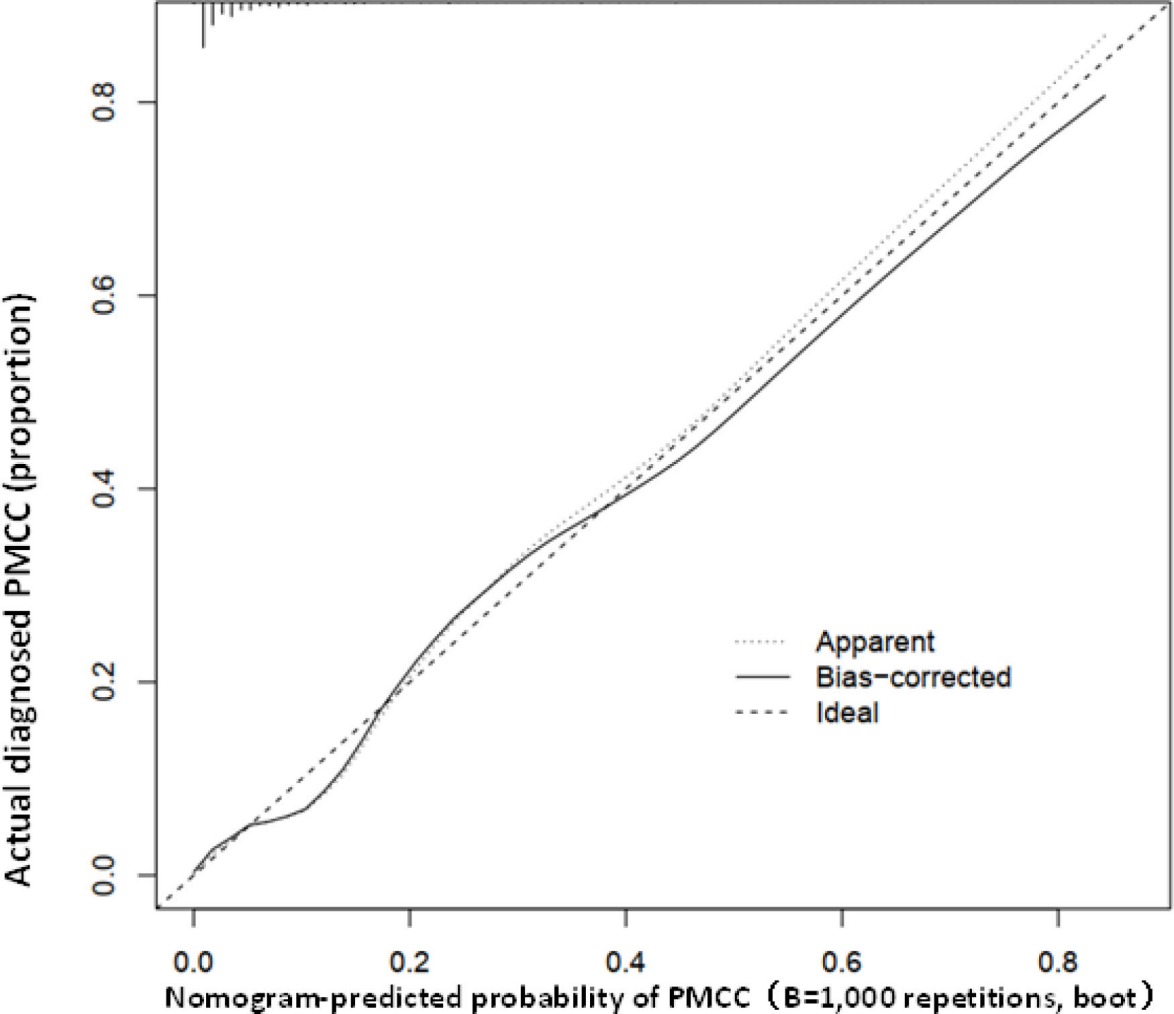
Receiver operating curve (ROC) of the nomogram.

**Figure 5:**
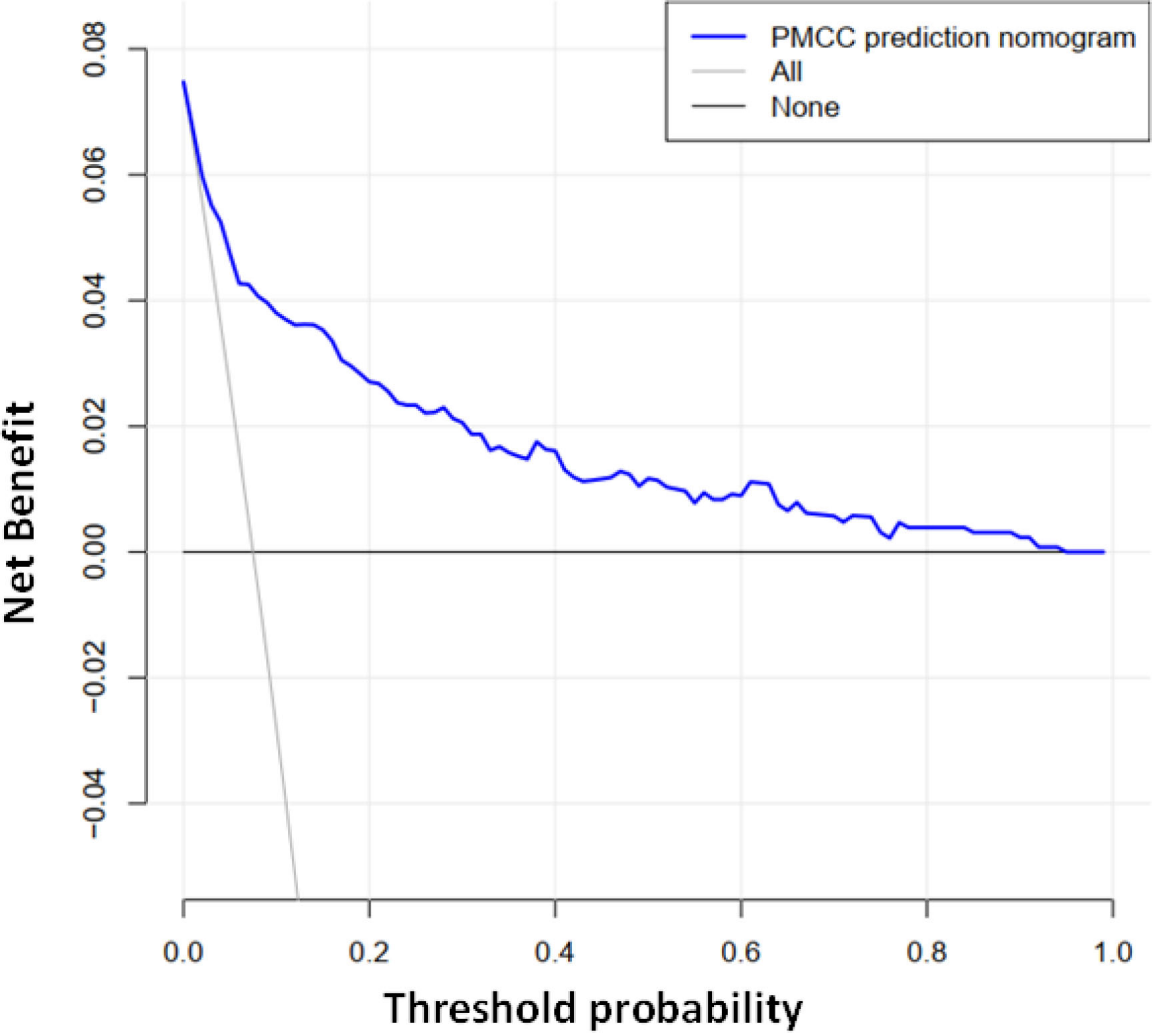
Decision curve analysis of the PMCC nomogram. The Y-axis represents the net benefit. The grey solid line represents the PMCC risk map. The black solid line signifies assumption of PMCC for all patients. The blue solid line indicates no PMCC assumption. DCA showed that under probability of PMCC threshold of 1–94%, using a nomogram to predict risk of PMCC was more beneficial than “all patient intervention” and “non-intervention” programs.

Using R software, the eight predictive variables screened *via* logistic regression were substituted into the nomogram prediction model (**Figure 2**). The outcome indicator was risk of PMCC, which was evaluated based on the total point score. ROC curve of the combined diagnosis was generated according to the results of software equation operation. AUC of the training set was 0.882 and C-index was 0.882 (95% CI: 0.845-0.919) (**Figure 3**), indicating good predictive ability of the model. After internal verification of the nomogram prediction model using the bootstrap method with 1 000 repeated samplings, the C-index value was 0.868, confirming high discriminative and predictive ability (**Figure 4**). The correction curve revealed good agreement between the prediction and actual results (**Figure 3**). Data from the decision curve analysis (DCA) are shown in the **Figure 5**. DCA findings suggest that with a predicted occurrence probability of PMCC in the range of 1 – 94% with the nomogram model, application of the nomogram to predict risk of PMCC is more beneficial relative to both “treat all patients” and “treat none” regimens.

## 4 Discussion

The peritoneum, a complex monolayer mesothelial cell structure (producing surface active phospholipids), is supported by the basement membrane and located on the connective tissue layer. The major function of peritoneum is to provide an effective barrier for preventing biological macromolecules, including tumor cells, from entering the cortex. Peritoneal dissemination refers to a series of events that begin with cancer cell shedding from the cancer nest into the peritoneal cavity, followed by their adherence to the mesothelial surface and, finally, invasion of the subperitoneal space for proliferation and angiogenesis (21). PMCC is a common advanced stage of colorectal cancer and often regarded as a pre-mortem state that reflects extensive spread of tumors. Among patients with colorectal cancer, about 10% progress to PMCC (22). Medical oncologists and gastroenterologists have reported poor prognosis of PMCC, with a median survival time of 6-9 months (23, 24). The group of Sugarbaker proposed that peritoneal cancer occurs due to local spread as a result of dialog between cancer cells and host molecules (25). Recent studies have shown that cytoreductive surgery (CRS) with hyperthermic intraperitoneal chemotherapy (HIPEC) improves survival in patients with PMCC (22.3 months vs 12.6 months) (26), while the UNICANCER PRODIGE 7 randomized clinical trial reports a median overall survival more than 41 months with or without HIPEC (41.7 months (95% CI 36.2–53.8) in the cytoreductive surgery plus HIPEC group and 41.2 months (35.1–49.7) in the cytoreductive surgery group (hazard ratio 1.00 [95.37% CI 0.63–1.58]; stratified log-rank p=0·99) (27),which is considered the only potentially curative option to achieve long-term survival (28). Until recently, the presence of peritoneal metastases (PM) originating from gastrointestinal tumors has been considered to indicate terminal disease. However, the emergence of improved systematic treatment, better understanding of prognostic factors, and the emergence of new local treatment modalities opened the door for the multimodal treatment of PM. These strategies, including radical surgery and thermoperitoneal chemotherapy (HIPEC), showed surprisingly promising results and prolonged the survival time of patients with peritoneal metastasis. Because the therapeutic effect of PMCC has been greatly improved in recent years and the therapeutic concept is mainly based on active intervention rather than palliative treatment (29), although from the current research status, there is still a long way to go to achieve revolutionary therapeutic effect. Therefore, considerable efforts should be made to identify patients with PC at the earliest stages before further dissemination of PMCC. However, current traditional imaging techniques cannot meet the clinical needs for accurate preoperative diagnosis of PMCC (30, 31). In an earlier retrospective study, ~23% colorectal cancer patients with peritoneal metastasis were misdiagnosed based on clinical and imaging profiles before surgery. Although molecular diagnostic techniques and CT colonography have developed rapidly in recent years, their application in practice remains a challenge due to the high false positive rates, lack of unified standards, and significant cost (32). Identification of independent risk factors for predicting PMCC is therefore of great clinical value. Our results suggest that age, tumor location, perimeter ratio, tumor size, pathological type, tumor invasion depth, CEA level, and gross tumor type are independent risk factors for PMCC. Peritoneal metastasis is caused by shedding of tumor cells from the primary lesion into the abdominal cavity, which could explain why colorectal cancer with deeper invasion is more likely to develop into peritoneal cancer. Our experiments showed that a positive correlation between depth of tumor invasion and PMCC. Earlier reports have documented a 10-fold increase in risk of PC after colorectal cancer invades the outer membrane (7, 33). Due to the abundance of blood vessels and lymphatic vessels in the plasma membrane of the intestinal wall, with greater tumor infiltration, a large number of active cancer cells may be separated after penetrating the plasma membrane and enter the abdominal cavity, forming free tumor thrombus that adheres to and degrades the extracellular matrix of the peritoneal cavity and is implanted in the peritoneal mesothelial tissue for proliferation, eventually leading to peritoneal metastasis (34). Our results are consistent with this theory. In addition, clinicopathological parameters, such as tumor size, distant organ metastasis and pathological type, are closely associated with peritoneal dissemination. Mucinous adenocarcinoma accounts for 6–20% of all colorectal cancer cases and peritoneal dissemination in mucinous adenocarcinoma is more intense than that in non-mucinous adenocarcinoma (4, 35). In general, in colorectal cancer with mucinous carcinoma pathotype, tumor invasion and dissemination, poor prognosis, and recurrence and peritoneal dissemination are more likely. Experiments by Negri et al. (36) showed that patients with mucinous carcinoma did not respond well to chemotherapy regimens based on 5-fluorouracil, oxaliplatin and irinotecan, which could partly underlie the high recurrence rates in these subgroups. Consistent with earlier findings, age over 60 was negatively correlated with risk of PMCC (OR value: 0.588 [95% CI: 0.177 to 0.890; p < 0.001]) in our patient population (7, 37). Statistical analysis of the primary location of tumors disclosed that risk of PMCC of tumors located in the rectum is low while that of tumors in the left and right colon regions is markedly higher. This finding may be attributed to the fact that primary tumors in the rectum are mainly located outside the peritoneal cavity, and therefore, tumor shedding and planting in the abdominal cavity would require a large tumor load. The results of Kerscher and van Gestel are partial consistent with our conclusion (4, 9), while for patients with sigmoid colon cancer, the conclusions arecontradictory to our findings. This discrepancy highlights the importance of the location of primary tumors of colorectal cancer for peritoneal metastasis. Other than anatomical location, the size of tumor infiltrating the intestinal circumference is one of the important factors affecting peritoneal metastasis. Notably, risk of peritoneal metastasis is positively correlated with size of primary tumor. In this study, the peritoneal metastasis rates of primary tumors based on size were 9.42% (72/692) in the intestinal cavity ≥1/2 diameter group and 4.629% (24/469) in the intestinal cavity ≤ 1/2 diameter group. This difference was statistically significant (χ2 = 10.343, P = 0.001). Tumors can grow to >1/2 the circumference of the intestinal cavity, suggestive of a rich blood supply that provides nutrition. The processes of tumor occurrence and development reflect proliferation and infiltration properties and tumor size indicates the stage of disease. Larger tumors are correlated with longer disease periods and deeper invasion. In addition, with treatment delay, tumor cells have sufficient time to form distant metastasis or micrometastasis lesions. The principle is similar in that greater tumor infiltration depth is associated with higher risk of PMCC. CEA > 10 ng/mL was included in the nomogram, potentially indicating that high serum CEA is related to risk of PMCC. Tumor load and possibility of peritoneal infiltration and metastasis were correlated with higher CEA index in serum. Increased serum CEA indicates a later stage of colorectal cancer and stronger proliferation of tumor cells (38), along with low tumor differentiation, poor pathological type and metastasis.

An effective prediction tool requires low cost, easy access to clinical data and relatively high accuracy. Here, we established a risk model by analyzing a large-capacity database, which led to the identification of eight risk indicators of PMCC. The purpose and practical value of any prediction model must be its applicability and relevance to clinical practice. The eight predictors included in this study, age, tumor location, perimeter ratio, tumor size, pathological type, tumor invasion depth, CEA level, and gross type are very easy to collect (**Figure 2**). According to the classification of various factors on the nomogram, we drew vertical lines above the horizontal points at each prediction factor, and the corresponding value is the score of this factor. Finally, the scores for these eight factors are added together to obtain a total score. A point can be found in the total score, and the vertical line is drawn again along this point. The corresponding value below is the risk probability of PMCC.On this basis, we developed a simple and easy-to-use nomogram to predict the possibility of PMCC, with a view to providing targeted assessment recommendations and interventions for patients.

In the training data set, the nomogram had good discrimination and calibration values, with AUC of 0.882. Decision-making curve analysis (DCA) showed that at a prediction probability of PMCC of 1–94%, the nomogram model was more beneficial for patients than the ‘whole patient treatment’ or ‘no patient treatment’ schemes. The bootstrapping method was additionally applied for internal validation of the nomogram. The scale map showed good consistency between prediction and observation results. A C-index of 0.868 was obtained, indicative of medium to high prediction ability, reasonable discrimination and acceptable scaling. The nomogram developed in our study incorporated not only CT-based features but also clinical data related to pathology, clinical symptoms, age, and tumor morphology, which are easy to obtain. Moreover, our nomogram showed good identification and calibration ability. Quantitative risk-predictive nomograms facilitate objective evaluation of risk of PMCC, thus helping to optimize individualized management of patients and reduce the pain and additional expenses caused by traumatic diagnosis. To this end, our collective findings support the utility of this newly developed nomogram as an effective tool for clinical treatment decision-making.

The current study has a number of limitations that should be taken into consideration. First, this is a retrospective analysis of single-center prospective databases, thus lacking prospective cohorts to validate accuracy and stability. Secondly, utilization of traditional imaging diagnosis technology inevitably causes measurement deviations. In addition, recent studies suggest that vascular and perineural invasion and a number of gene mutations are related to specific metastatic organs in CRC. For example, BRAF mutations are associated with peritoneal dissemination (39). Yaeger et al. (40) examined the genomic map of 1134 cases of colorectal adenocarcinomas and found that there is a high degree of genomic consistency between primary tumors and metastatic lesions, indicating that most mutations develop in primary tumors, not at metastatic sites. In their analysis, mutations in NRAS, KRAS, BRAF, and APC were all associated with poor survival, confirming the known adverse effects of these mutations (41). In addition, mutations affecting RAS/RAF and other genes, including PIK3CA, PTEN, AKT1, SMAD2, and SMAD4 appear to be associated with a high risk of peritoneal metastasis. Other studies reported that the incidence of PM in patients with tumors carrying the V600E BRAF mutation was three times higher than that in tumors with wild-type BRAF (42, 43). Mutations in RAS, high PCI, and lymph node status were identified as specific risk factors for peritoneal recurrence. These new findings emphasize the heterogeneity of colorectal metastasis. Given the importance of these mutations it is regrettable that we could not include them in the predictive nomogram. However, data about the factors included in the nomogram can be collected immediately, or very shortly after surgery. In practice the results of tests investigating the presence of mutations affecting RAS/RAF, or other relevant genes are unlikely to be available at this point in time. Furthermore, mutation testing is currently not carried out in the majority of patients. No routine detection of RAS/RAF mutations was performed in early patients, partly because these are not routine examination tools. Thus, we were not able to incorporate information on gene mutations into the analysis and statistical modelling of biological behavior. Finally, although the robustness of our nomogram was extensively validated internally through bootstrap tests, no external verification was performed, and its generalized application for other regions and countries may be questionable. Despite these limitations, we believe that our nomogram prediction model provides a strong reference for development of individualized treatment regimens for PMCC patients. Further studies are warranted to obtain data that can be integrated to accurately identify the patients requiring additional chemotherapy or radiotherapy and, conversely, avoid overtreatment in other groups.

## 5 Conclusion

We constructed a robust nomogram using clinical variables associated with PMCC, which showed good predictive ability. This nomogram may be utilized as a tool to strengthen early diagnosis of PMCC and aid in optimal development of individualized treatment plans by clinicians in the future. However, prior to clinical application, studies on more multi-center databases are required for external validation to verify the prediction accuracy and generalization ability of the newly developed nomogram.

## Data availability statement

The raw data supporting the conclusions of this article will be made available by the authors, without undue reservation.

## Ethics statement

All patients provided written informed consent for information storage in the hospital database of the First Affiliated Hospital of Guangxi Medical University.

## Author contributions

X-QS was responsible for study design, data acquisition and analysis, and manuscript writing. X-QS and Z-XH performed bioinformatics and statistical analyses. X-QS, Z-XH, Z-XL and Q-YK were responsible for collecting clinical samples. X-QS and Z-HH prepared the figures and tables for the manuscript. SZ were responsible for the integrity of the entire study and manuscript review. All authors contributed to the article and approved the submitted version.

## Conflict of interest

The authors declare that the research was conducted in the absence of any commercial or financial relationships that could be construed as a potential conflict of interest.

## Publisher’s note

All claims expressed in this article are solely those of the authors and do not necessarily represent those of their affiliated organizations, or those of the publisher, the editors and the reviewers. Any product that may be evaluated in this article, or claim that may be made by its manufacturer, is not guaranteed or endorsed by the publisher.
